# Oxidative Stress and Liver Morphology in Experimental Cyclosporine A-Induced Hepatotoxicity

**DOI:** 10.1155/2016/5823271

**Published:** 2016-05-19

**Authors:** Agnieszka Korolczuk, Kinga Caban, Magdalena Amarowicz, Grażyna Czechowska, Joanna Irla-Miduch

**Affiliations:** ^1^Department of Clinical Pathomorphology, Medical University, Jaczewskiego 8, 20-950 Lublin, Poland; ^2^Department of Gastroenterology with Endoscopic Unit, Medical University, Jaczewskiego 8, 20-950 Lublin, Poland

## Abstract

Cyclosporine A is an immunosuppressive drug used after organ's transplantation. The adverse effects on such organs as kidney or liver may limit its use. Oxidative stress is proposed as one of the mechanisms of organs injury. The study was designed to elucidate CsA-induced changes in liver function, morphology, oxidative stress parameters, and mitochondria in rat's hepatocytes. Male Wistar rats were used: group A (control) receiving physiological saline, group B cyclosporine A in a dose of 15 mg/kg/day subcutaneously, and group C the CsA-vehicle (olive oil). On the 28th day rats were anesthetized. The following biochemical changes were observed in CsA-treated animals: increased levels of ALT, AST, and bilirubin in the serum, statistically significant changes in oxidative stress parameters, and lipid peroxidation products in the liver supernatants: MDA+4HAE, GSH, GSSG, caspase 3 activity, and ADP/ATP, NAD^+^/NADH, and NADP^+^/NADPH ratios. Microscopy of the liver revealed congestion, sinusoidal dilatation, and focal hepatocytes necrosis with mononuclear cell infiltration. Electron microscope revealed marked mitochondrial damage. Biochemical studies indicated that CsA treatment impairs liver function and triggers oxidative stress and redox imbalance in rats hepatocytes. Changes of oxidative stress markers parallel with mitochondrial damage suggest that these mechanisms play a crucial role in the course of CsA hepatotoxicity.

## 1. Introduction

Cyclosporine A (CsA) belongs to calcineurin inhibitors used in patients after kidney, liver, heart, lung, and heart-lung transplants for graft-versus-host disease (GVHD) prophylaxis [1, 2].

Moreover, CsA is used to treat the majority of autoimmune diseases [3], in dermatology to treat psoriasis, autoimmune dermatitis, or chronic idiopathic urticaria [4, 5].

The major adverse side effect of CsA is acute and chronic nephrotoxicity.

CsA can cause metabolic and electrolyte disorders, that is, weight gain, hyperglycaemia, hyperlipidaemia, hypercalcaemia, and hypomagnesaemia [6].

Experimental studies and clinical observations reveal that CsA can lead to drug-induced liver injury (DILI). In CsA-induced liver injury, functional and morphological changes are observed. The functional changes include elevated serum levels of liver transaminases and alkaline phosphatase, cholestasis, hyperbilirubinemia, increased production of bile salts, and impaired secretion of lipids [7–9].

The morphological changes observed in experimental animals receiving CsA include impaired trabecular structure, hepatic sinus congestion and widening, activation of the Kupffer cells, passive congestion and oedema of portal tracts, mild mononuclear cell infiltrations within portal tracts, and degenerative changes in the hepatocytes including their focal necrosis [10–12].

The mechanisms of CsA-induced liver injury involve the development of hypermetabolic state in the liver [13] and inhibition of ATP-dependent transport of bilirubin and bile salts through the hepatocyte canalicular membranes as well as of bile secretion [14, 15]. The use of antioxidants in experimental animals exposed to CsA reduces liver functional and morphological damage [11, 12, 16, 17], which suggests the involvement of oxidative stress as one of the mechanisms of hepatotoxicity.

The aim of the present study was to evaluate the function and morphology of the liver in animals receiving a cumulative dose of CsA. We focused on the correlation between changes in the selected oxidative stress parameters and morphological and ultrastructural changes in hepatocytes.

## 2. Material and Methods

Adult male Wistar rats weighing 250–300 g were housed in a temperature-controlled environment with an alternating cycle of 12 h light and dark. They were on a low-sodium diet and had free access to water. The experimental protocols were conducted according to the guidelines of Institutional Animal Ethics Committee (IAEC) of the Medical University, Lublin.

The animals were divided into three groups (A, B, and C) (with 8 animals in each group):

 A: control, NaCl 1 mL/kg/day, subcutaneously.
 B: vehicle, olive oil 1 mL/kg/day, subcutaneously.
 C: CsA, 15 mg/kg/day in olive oil, subcutaneously.

CsA, NaCl, and olive oil doses and way of administration were established according to previous studies [10, 16]. Animals were weighed daily while receiving treatment for 28 days. On the 29th day of an experiment all animals were anesthetized with pentobarbitone (Morbital, Biowet, Poland) and blood samples and liver specimens from the left and right lobe were obtained for biochemical, histological, and ultrastructural analyses.

### 2.1. Measurement of Liver Function

Serum levels of AST, ALT, and bilirubin were measured using the commercially available diagnostic Cormay kits (Cormay Diagnostics SA, Poland).

### 2.2. Biochemical Studies

The liver samples were homogenised in 20 mM phosphate buffer (pH 7.4), 0.5 g tissue in 2 mL. The homogenisation was made in cold-water bath (4°C) at 4000 rpm using a Teflon pestle homogeniser (Glas-Col, USA) for 3 min. The homogenate was centrifuged at 15 000 rpm for 20 min and the obtained supernatant was used for further biochemical studies. All spectrophotometric methods were performed using a microtiter plate reader (PowerWaveXS, BioTek, USA).

*GSH, GSSG, and GSH/GSSG*. Reduced (GSH) and oxidized (GSSG) glutathione determination was conducted using commercial kit BIOXYTECH GSH/GSSG-412 (*Oxis*Research, USA). The principle of the procedure is based on simultaneous determination of GSH and GSSG in two separate tubs. To determine GSSG, 1-methyl-2-vinylpyridinium trifluoromethane-sulfonate (M2VP) was used at a level that rapidly and completely scavenges GSH but does not interfere with the glutathione reductases that in turn reverse GSSG to GSH in the next step of procedure. Subsequently the procedure is common for both parameters. GSH in separate tubs (created or native) is extracted and reacts with Ellman's reagent (5,5′-dithiobis-2-nitrobenzoic acid) forming a colour product with the maximum of absorbance at 412 nm. The concentrations of GSH and GSSG were rewritten from the separate calibration curves. 

*Lipid Peroxidation Products*. The commercial kit BIOXYTECH LPO-586 (*Oxis*Research, USA) was used to measure malondialdehyde (MDA) and 4-hydroxyalkenals (4HAE) as indicators of lipid peroxidation. The method is based on the reactions between MDA and 4HAE with N-methyl-2-phenylindole at temperature of 45°C for 60 minutes, in which a stable chromophore with maximal absorbance at 586 nm is yielded. The procedure was conducted according to the manufacturer's description and the concentration of MDA+4HAE in tested samples was calculated from the formula of calibration curve *y* = 0.0896*x* − 0.008. The results were expressed in nmol/g liver tissue. 

*NAD* ^*+*^ */NADH and NADP* ^*+*^ */NADPH Ratios*. The NAD(P)^+^ and NAD(P)H levels were measured using BioChain NAD^+^/NADH assay kits according to the manufacturer's instructions (BioChain, Hayward, CA). The principles of assay kits are based on a glucose dehydrogenase cycling reaction, in which the tetrazolium dye (MTT) is reduced by NAD(P)H in the presence of phenazine methosulfate (PMS). The intensity of the reduced product colour, measured at 565 nm, is proportionate to the NAD^+^ concentration in the sample. The standards attached to the kits were used to prepare the calibration curves needed to calculate NAD(P)^+^/NAD(P)H ratios. 

*ADP/ATP Ratio*. The tissue supernatant ADP/ATP ratio was calculated on the basis of fluorescence (luminescence) intensity measurement (BioVision kit, USA) using a fluorescence microplate reader Victor3V (Perkin Elmer, Finland). 

### 2.3. Morphologic Studies

Liver samples were fixed in 10% buffered formalin and embedded in paraffin. After dewaxing 4 *μ*m sections were stained with haematoxylin and eosin (H+E). All slides were evaluated under light microscopy (Olympus BX45) by one pathologist who assessed liver morphology. A minimum of 20 cortical fields was examined in each biopsy (magnification ×100).

### 2.4. Electron Microscopy

Tissue sections for electron microscopic examinations were fixed in 4% formaldehyde and 1% glutaraldehyde and processed to epoxy resin. Ultrathin samples were stained with uranyl acetate and lead citrate and examined with a Jeol-JEM 1011 electron microscope.

### 2.5. Statistical Analysis

Results were presented as mean ± SEM. Data were analysed by one way analysis of variance (ANOVA) followed by Duncan's multiple range test and Student's *t*-test using a statistical software package (STATISTICA v.8.0 StatSoft, Poland). *p* value < 0,05 was considered statistically significant.

## 3. Results

### 3.1. Liver Function

CsA administration resulted in decreased liver function measured by serum levels of AST, ALT, and bilirubin when compared with the control group (Table 1). The results show statistically significant differences in all examined parameters in animals of group B comparing with animals of control group (*p*< 0,05): AST was 62,4 in group B versus 25,09 in group A, ALT 28,35 (group B) versus 7,76 (group A), and bilirubin 1,06 (group B) versus 0,42 (group A).

### 3.2. Microscopic Changes

**Figure 1:**
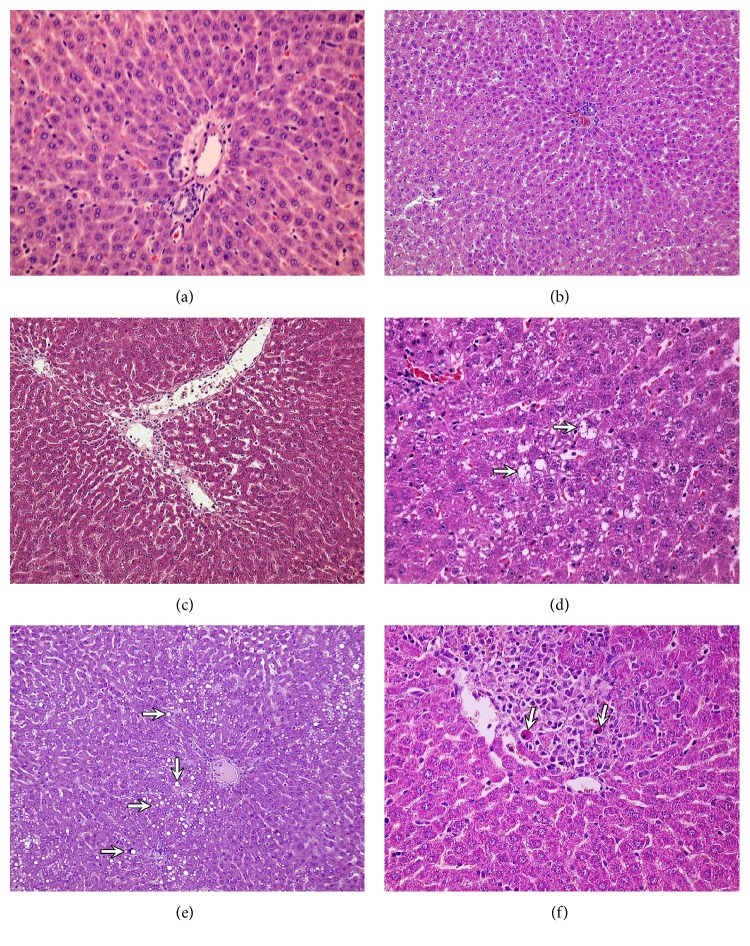
Light microscope, H+E. (a) Control group. Liver architecture within normal limits. ×400. (b) Olive oil group. Mild passive hyperaemia within the central vein. ×200. (c)–(f) CsA group. (c) Passive hyperaemia within central veins with dilatation of hepatic sinusoids. ×200. (d) Vacuolar degeneration of the cytoplasm of hepatocytes (arrows). ×400. (e) Focal micro- and macrovesicular steatosis (arrows). ×200. (f) Focal liver cells necrosis accompanied by mononuclear inflammatory infiltrates. Single eosinophilic cells with signs of apoptosis present (arrows). ×400.

No prominent morphologic changes were seen in the liver of group receiving olive oil or control animals (Figures 1(a) and 1(b)). Liver samples collected from CsA-treated animals (group B) showed several morphological changes. Lobular architecture of the liver was preserved. Passive hyperaemia within central veins with dilatation of hepatic sinusoids was observed (Figure 1(c)). Hepatocytes presented vacuolar degeneration of their cytoplasm (Figure 1(d)) and focal micro- and macrovesicular steatosis (Figure 1(e)). Foci of liver cell necrosis accompanied by mononuclear inflammatory infiltrates were noticed with the presence of single cells with pyknotic nuclei and strongly eosinophilic cytoplasm (Figure 1(f)).

### 3.3. Ultrastructural Changes

**Figure 2:**
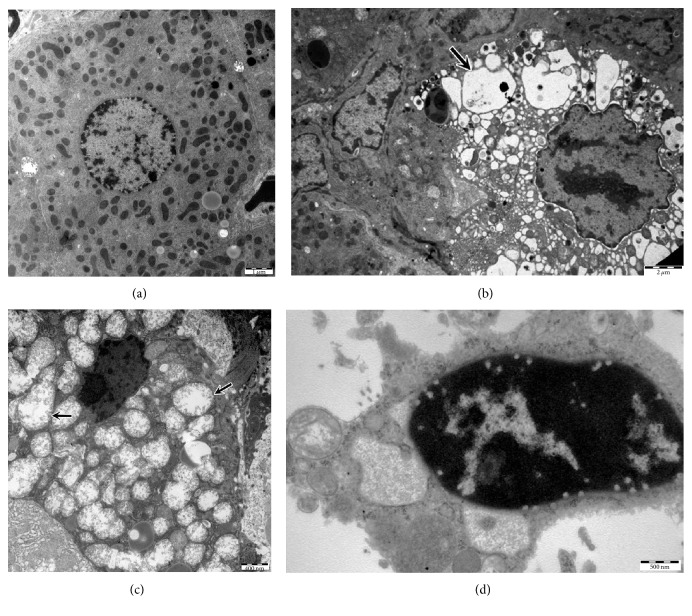
Electron microscope. (a) Control group. Hepatocyte with normal ultrastructural appearance. ×8000. (b–d) CsA group. (b) Hepatocyte with dilatation of endoplasmic reticulum with the formation of different sized vacuoles (arrow). ×8000. (c) Marked swelling of mitochondria with the presence of giant mitochondria (arrows) with disruption of mitochondrial cristae. Shrunken nucleus with condensed chromatin present. ×20000. (d) Apoptotic cell with markedly condensed chromatin and shrunken cytoplasm. ×60000.

Electron microscope examination did not reveal pathologic changes within the hepatocytes of group receiving olive oil or control animals (Figure 2(a)). In animals treated with CsA, vacuolization of hepatocytes seen under a light microscope corresponded to dilatation of endoplasmic reticulum with the formation of different sized vacuoles seen in electron microscope (Figure 2(b)). Ultrastructural examination of these cells revealed also marked swelling of mitochondria with the presence of giant mitochondria in some cells (Figure 2(c)). In most of these organelles, the disruption and loss of the inner membrane and the cristae were observed (Figure 2(c)). Moreover, the formation of autolysosomes containing mitochondrial material was observed. Mitochondrial damage was observed approximately in 50 to 60% of hepatocytes of animals receiving CsA. Some of nuclei were shrunken with some of them performing karyorrhexis (Figures 2(b) and 2(c)). Single apoptotic cells with markedly condensed chromatin and shrunken cytoplasm were present (Figure 2(d)).

### 3.4. LPH, GSH, and GSSG

Treatment of animals with CsA alone produced a significant increase in hepatic LPH (3,13 nmol/g in controls to 12,68 nmol/g in CsA animals) and GSSG levels (0,38 nmol/g in controls to 1,33 nmol/g in CsA animals) as well as a significant decrease in hepatic GSH levels (6,65 nmol/g in controls to 3,3 nmol/g in CsA animals) compared with controls. The results showed statistically significant differences in all examined parameters in animals of group B comparing with animals of control group (*p*< 0,05), Table 2.

### 3.5. NADP^+^/NADPH, NAD^+^/NADH, and ADP/ATP Ratios

CsA induced a significant increase in NADP^+^/NADPH ratio (from 0,72 in controls to 3,48 in CsA animals) and a decrease in NAD^+^/NADH ratio (from 5,83 in controls to 1,78 in CsA animals), Table 2. The results showed statistically significant differences in all examined parameters in animals of group B comparing with animals of control group (*p*< 0,05). The mean ADP/ATP ratio was significantly higher in CsA-treated animals (4,95) compared with controls (0,8).

### 3.6. Caspase 3 Activity

Caspase 3 activity in the liver was significantly increased (*p*< 0,05) in animals receiving CsA when compared with controls (Table 2). It increased from 99,93% in controls to 232,22% in CsA group.

## 4. Discussion

In the present study assessing the effects of CsA on liver injury in animals, changes in liver function parameters, microscopic and ultrastructural lesions in the hepatocytes, and changes in oxidative stress parameters were analysed.

The essential adverse effect of CsA is nephrotoxicity, yet there are also reports describing damage to other organs or systems, such as the heart, central nervous system, and testicles [6, 18–21]. Moreover, clinical and experimental studies have demonstrated impaired liver function and morphology [10–12, 22, 23].

Our findings revealed that administration of CsA increased levels of AST, ALT, and bilirubin and these findings are consistent with the results of experimental studies of other authors, which show that elevated levels of these parameters evidence functional liver damage [8, 23–27].

Drug-induced liver injury can develop in the form of acute drug-induced hepatitis or cholestatic hepatitis [28, 29]. Toxic effects of drugs can cause degenerative changes in the hepatocytes, including their necrosis (paracetamol, bendazac, CsA, carbon tetrachloride, and ethionine), steatosis (tetracycline and ticlopidine), or cholestasis (methapyrilene and naphthyl isothiocyanate) [30]. Three major mechanisms of drug-induced liver injury have been implicated: (1) direct cell injury, (2) inhibition of mitochondrial beta-oxidation and the mitochondrial respiratory chain, and (3) immunologic reactions [31]. The symptoms of toxic action of drugs observed in histopathological examination include hepatocyte degeneration or necrosis, inflammatory infiltrates in the portal tracts, Kupffer cells, or stellate cell activation [32–34]. The mechanisms explaining drug-induced liver injury include mitochondrial damage and oxidative stress [29, 35–39].

In the present study, the light and electron microscopy findings demonstrated significant differences in liver morphology in rats receiving cyclosporine, as compared to the control group. Congestion and widening of hepatic sinuses, activation of Kupffer cells, passive congestion and swelling in the portal tracts, mild inflammatory mononuclear cell infiltrates within the portal tracts, and hepatocyte degenerative changes, vacuolar degeneration and steatosis, were present. Similar findings have also been described in other experimental studies [10–12, 24, 27, 40] demonstrating focal necrosis of hepatocytes and the presence of apoptotic cells [10–12, 40].

Drug toxicity-induced changes at the ultrastructural level predominantly regard the mitochondria [29, 36, 37]. Due to their characteristic structure and function different from those of other cell elements, the mitochondria are the key target of drug toxicity [29]. Oxidative stress mostly affects these cell structures. The evaluation of mitochondrial ultrastructure in correlation with the assessment of the selected oxidative stress parameters was an important element of our study.

The role of oxidative stress in chronic CsA treatment has been examined in several studies [41–47]. CsA induces intramitochondrial Ca^++^, increases oxidative stress and ROS production, and inhibits mitochondrial glucose metabolism (the Krebs cycle and oxidative phosphorylation) and ATP production [6]. It is postulated that CsA is an uncoupler and inhibitor of the mitochondrial transport system. CsA-induced ROS generation is activated by NADPH oxidase, xanthine oxidase, cytochrome P450 CsA metabolism, or decreased intracellular antioxidant systems [48]. The ROS level in the kidney is dose-related [45, 49]. Increase in ROS results in lipid peroxidation and increases its products such as MDA. Moreover, CsA treatment reduces GSH, an important antioxidant [40, 41, 50–53]. Reduced glutathione (GSH) converts lipid peroxides to nontoxic products, thus maintaining the integrity of the mitochondria and cell membranes. GSH is converted by glutathione peroxidase to GSSG in the glutathione redox cycle. Regeneration of GSH by glutathione reductase uses NADPH. In present study, significant increases in MDA and decreases in GSH levels in animals treated with CsA were observed; our results are consistent with those reported in other studies [12, 23, 24, 54, 55]. It may be speculated that marked decreases in GSH not only were produced by engagement in ROS reduction but could also result from impaired regeneration by glutathione reductase.

Lipid peroxidation products, MDA and mainly 4HAE, decrease the ADP- and NADH-dependent mitochondrial respiratory chain [56], which results in disturbances in membranous Na^+^K^+^ ATPase activity and decreases in the main mitochondrial product: ATP. To assess mitochondrial phosphorylation and redox state, ADP/ATP, NAD^+^/NADH, and NADP^+^/NADPH ratios were investigated. CsA-treated animals showed a significant increase in the ADP/ATP ratio when compared with controls. Impaired mitochondrial respiration could be one of the possible explanations. These findings correlate with decreases in NAD^+^/NADH ratio. Decreased regeneration of NAD^+^ and/or increased NADH^+^ might result from respiratory chain impairment. Oxidative stress increases NAD^+^ and ATP consumption and promotes PARP-1 (nuclear enzyme: poly, ADP-ribose, and polymerase-1) dependent cell death [57]. Decreased NAD^+^/NADH ratios promote further mitochondrial damage and can lead to cell death.

Increases in NADP^+^/NADPH ratio were noticed in animals treated with CsA. It could be associated with consumption of NADPH in antioxidative mechanisms. De Hornedo et al. [58] in their* in vitro* study have found that CsA causes dysfunction of enzymes responsible for NADPH production, such as mitochondrial dehydrogenases. NADPH is also a substrate for NADPH oxidase in ROS production. We cannot exclude that the increased NADP^+^/NADPH ratio observed in this protocol is the result of NADPH oxidase stimulation and dysfunction of peroxisome dehydrogenases and in consequence a decrease in NADPH. Furthermore, it cannot be excluded that an increase in NADP^+^/NADPH ratio could be associated with increased activity of glutathione reductase at the beginning of the experiment. Decreased GSH and increased GSSG levels seem to support this hypothesis.

Another observation of our study was a substantial increase in caspase 3 activity in the group receiving CsA, as compared to the control group. Substantially increased caspase 3 activity in the liver of rats given CsA compared to controls has also been demonstrated by Wolf et al. [59]. Moreover, similar changes have also been observed in kidneys of rats receiving cyclosporine [60]. In the case of apoptosis, released cytochrome c binds cytoplasmic scaffold (apaf-1) and procaspase 9, forming the apoptosome. The apoptosome activates procaspase 9. The above process required energy derived from ATP. When some mitochondria remain intact and their function consisting in ATP production is normal, activated procaspase 9 and other proapoptotic proteins activate caspase 3, which uncouples specific cell proteins and subsequently activates procaspases 6, 7, and 2, which uncouple some other proteins [61, 62]. The above changes induce programmed cell death, that is, apoptosis [63]. The initiation of apoptosis is thought to be triggered by increased permeability of the outer mitochondrial membrane and escape of cytochrome c from the mitochondrion to the cytosol.

Since mitochondria are one of the main physiologic sources of ROS, we have also focused on their structural appearance. Ultrastructural findings showed swelling of mitochondria in the hepatocytes that differed in size with rupture of their cristae, the inner and outer membrane present in some of them. Degradation of mitochondria and autolysosomes containing the mitochondrial material were also observed. Single apoptotic cells were present. Similar observations were described in previous studies [57, 64, 65]. Structural damage to mitochondria seems to result from their impaired function. De Hornedo et al. [58] have documented that CsA produces a depolarization of mitochondrial membranes that parallels with ROS production. CsA promotes a caspase-independent release of proapoptotic cytochrome c and Smac/Diablo from mitochondria [64]. Increased release of cytochrome c to the cytosol has also been described [65], suggesting that CsA opens the so-called mitochondrial permeability transition pores (MPTP) created at sites of contact of the inner and outer mitochondrial membranes.

Apoptosis is associated with condensation of the nucleus and cytoplasm as well as cytoplasm fragmentation without the loss of cell membrane integrity. Thus, it cannot be excluded that apoptosis was one of the mechanisms of necrosis of single hepatocytes observed by us. This hypothesis of mitochondrial degradation with release of proapoptotic cytochrome c could partially explain the presence of single apoptotic cells in our ultrastructural studies.

Oxidative stress as the possible mechanism of CsA hepatotoxicity was investigated by studies with the use of antioxidants [10, 11, 23, 25, 26, 62, 66–68]. Their findings have demonstrated hepatoprotective effects of melatonin [11, 16], L-arginine [24], taurine [23], quercetin, vitamin E [69], n-acetylcysteine [68], and S-adenosylmethionine [22].

It is known that the mitochondrion is a major intracellular source of ROS. As major ROS generators, mitochondria are targets of high ROS exposure with consequences, such as oxidative damage to mitochondrial DNA (mtDNA) [63]. Considering high number of mitochondria in hepatocytes and main functions of mitochondria, such as energy production and cell death regulation [70, 71], it can be supposed that damage to these organelles is a consequence of elevated ROS. Due to oxidative stress, mitochondria become more susceptible to the action of drugs; mitochondrial damage is a relevant mechanism of toxicity of drugs, including CsA [10, 63, 72]. While elevated ROS associated mtDNA damage has been implicated in cell apoptosis, the precise mechanism whereby mtDNA damage mediates apoptotic signaling is incompletely understood [63]. The impaired liver function and impaired morphology of mitochondria observed by us at the ultrastructural level suggest that oxidative stress triggered the damage to these cytoplasmic organelles and that this mechanism plays an important role in CsA-induced liver injury. Further experimental and molecular studies including cell cultures studies and use of selected antioxidants are considered by us to support this hypothesis.

## Figures and Tables

**Table 1:** Serum levels of AST, ALT, and bilirubin in all experimental groups.

	NaCl (A)	CsA (B)	Olive oil (C)
AST [U/L]	25,09 ± 3,01	62,4 ± 5,36^*∗*^	25,11 ± 3,36
ALT [U/L]	7,76 ± 0,98	28,35 ± 5,12^*∗*^	7,95 ± 1,26
BIL [mg/dL]	0,42 ± 0,038	1,05 ± 0,09^*∗*^	0,46 ± 0,05

Values are mean ± SEM. ^*∗*^A versus B and B versus C and *p* < 0,05.

**Table 2:** Hepatic levels of GSH, GSSG, LPH, NADP^+^/NADPH, NAD^+^/NADH, ADP/ATP, and caspase 3 in all experimental groups.

	NaCl (A)	CsA (B)	Olive oil (C)
GSH [nmol/g]	6,65 ± 0,64	3,3 ± 0,48^*∗*^	6,37 ± 0,74
GSSG [nmol/g]	0,38 ± 0,07	1,33 ± 0,23^*∗*^	0,4 ± 0,09
MDA+4HAE [nmol/g]	3,13 ± 0,94	12,68 ± 1,26^*∗*^	3,41 ± 0,56
NAD^+^/NADH	5,83 ± 0,81	1,78 ± 0,55^*∗*^	5,54 ± 1,16
NADP^+^/NADPH	0,72 ± 0,14	3,48 ± 0,49^*∗*^	0,74 ± 0,14
ADP/ATP	0,8 ± 0,24	4,95 ± 0,64^*∗*^	0,8 ± 0,24
Caspase 3 [%]	99,93 ± 18,94	232,22 ± 21,44^*∗*^	103,13 ± 18,94

Values are mean ± SEM. ^*∗*^A versus B and B versus C and *p* < 0,05.
